# Differential effects of age on circulating and splenic leukocyte populations in C57BL/6 and BALB/c male mice

**DOI:** 10.1186/1742-4933-5-1

**Published:** 2008-02-11

**Authors:** Lesya M Pinchuk, Nikolay M Filipov

**Affiliations:** 1 Department of Basic Sciences, College of Veterinary Medicine, Mississippi State University, Mississippi State, MS, USA; 2 Center for Environmental Health Sciences, Department of Basic Sciences, College of Veterinary Medicine, Mississippi State University, Mississippi State, MS, USA

## Abstract

**Background:**

Despite several reports on age-related phenotypic changes of the immune system's cells, studies that use a multipoint age comparison between the specific and innate immune cell populations of prototypical Th1- and Th2-type polarized mouse strains are still lacking.

**Results:**

Using a multipoint age comparison approach, cells from the two major immune system compartments, peripheral blood and spleen, and flow cytometry analysis, we found several principal differences in T cell and professional antigen presenting cell (APC) populations originating from a prototypical T helper (Th) 1 mouse strain, C57BL/6, and a prototypical Th2 strain, BALB/c. For example, regardless of age, there were strain differences in both peripheral blood mononuclear cells (PBMC) and spleens in the proportion of CD4+ (higher in the BALB/c strain), CD8+ T cells and CD11b+/CD11c+ APC (greater in C57BL/6 mice). Other differences were present only in PBMC (MHC class II + and CD19+ were greater in C57BL/6 mice) or differences were evident in the spleens but not in circulation (CD3+ T cells were greater in C57BL/6 mice). There were populations of cells that increased with age in PBMC and spleens of both strains (MHC class II+), decreased in the periphery and spleens of both strains (CD11b+) or did not change in the PBMC and spleens of both strains (CD8+). We also found strain and age differences in the distribution of naïve and memory/activated splenic T cells, e.g., BALB/c mice had more memory/activated and less naive CD8+ and CD4+ T cells and the C57BL/6 mice.

**Conclusion:**

Our data provide important information on the principal differences, within the context of age, in T cell and professional APC populations between the prototypical Th1 mouse strain C57BL/6 and the prototypical Th2 strain BALB/c. Although the age-related changes that occur may be rather subtle, they may be very relevant in conditions of disease and stress. Importantly, our data indicate that age and strain should be considered in concert in the selection of appropriate mouse models for immunological research.

## Background

Recent studies indicate that the immune system undergoes gradual age-related shifts in cell populations, which lead to functional changes of the immune responses. The compensatory modulations, including lymphocyte alterations, were recently defined as immunosenescence. This is a complex process of multiple reorganizational and developmentally regulated changes rather than a simple unidirectional decline in all immune functions [1,2]. Nevertheless, for the most part, the activity of the immune system declines with age, with the most pronounced alterations found in cell-mediated immunity (CMI), especially in the T cell functions, which are related to thymic involution [3-8]. Although decline in adaptive immunity represents a major problem for the aged, evidence accumulated within the last decade indicates that aging also has a profound impact on innate immunity [9].

Despite the maintenance of normal CD3+ cell numbers with age, there is a considerable decrease in CD4- and CD8-mediated responses [10,11]. One major reason for CMI decreases with age is the substantial reduction in the representation of naïve T lymphocytes with a concomitant increase in memory T cells. This is a consequence of compensatory homeostatic proliferation in response to the reduced numbers of naïve cells and the influence of cumulative exposure to pathogens and environmental antigens [12,13]. A second key age-related change is the alteration of the activation potential of memory T cells [14,15], leading to hyporesponsivity [16]. Also, there is an increased oligoclonal expansion of nontransformed T cell populations [17,18].

Additional shifts have also been documented in other cells of the ageing immune system, such as changes in the levels of CD4+ cells and proportion of CD4+/CD8+ populations in peripheral tissues [19,20]. The most consistent finding associated with a repressed immune response has been a decrease in the proportion of CD4+ T cells [21,8]. The appearance of multiple CD8+ T cell clonal expansions is one of the most dramatic qualitative changes in the memory cell population during ageing [22].

There is an agreement that ageing results in perturbation of peripheral blood B cells in two important ways. First, the number of newly made B cells that migrate to the spleen from the bone marrow is reduced [23,24]. Second, there is an accumulation of B lineage cells in the splenic compartments [23,24]. Many of these effects may be a consequence of functional defects intrinsic to the B cells [25,26], but others may be secondary to age-related changes in CD4+ T cells. Indeed, aged CD4+ T cells are less efficient at inducing germinal center formation and promoting somatic hypermutation [27,25]. This possibly reflects a shift from T helper 1 cell (Th1) to Th2-type cytokine patterns associated with age in mice and humans [28]. The factors that determine whether a proliferating CD4+ T cell in mice and humans will differentiate into a Th1 or Th2 cell are not fully understood. However, the consequences of inducing Th1 versus Th2 profiles are profound: the selective production of Th1 cells leads to CMI, whereas the production of predominantly Th2 cells provides humoral immunity. Recent studies have shown that the interaction of the most powerful APC, dendritic cells (DC), directly with pathogens through toll-like receptor (TLR)-dependent mechanisms or with innate lymphocytes represents a major control mechanism for adaptive immunity, including Th polarization [29-31].

Age-related shifts in cell population profiles may lead to a different humoral or cellular immune response bias in mice. In addition to age, genetics play a major role in the shaping of the immune response. Thus, the CD4/CD8 ratio, B cell apoptosis, and pre-B cell expansion are under genetic control in mice and humans [32-34]. Significant strain differences have been found in hematopoietic progenitor cell functions between B6, BALB and D2 mice [33-35]. Multiple reports suggest a preferential bias for the C57BL/6 mouse strain to develop Th1-type response, whereas the BALB/c strain is biased towards a Th2-type cytokine polarization to some infectious agents, including *Leishmania major*, *Pseudomonas aeruginosa*, and *Porphyromanas gingivalis *[36-40].

Previous studies have focused on the genetic basis of strain differences in peripheral blood cell populations [41]. However, the other major component of the immune system, the spleen, has been overlooked. Moreover, effects of ageing on APC have not received much attention. Despite several reports on age-related changes in the cells of the immune system (discussed in [2]), comprehensive studies that use a multipoint age comparison between the leukocyte populations of mouse strains that develop different immune responses, are still lacking. Hence, our objective was to perform a detailed side-by-side comparison of the age-related changes in peripheral blood and splenic T cell and professional APC populations in prototypical Th1 and Th2 mouse strains, C57BL/6 and BALB/c, respectively.

## Results

### Body and spleen weight changes with age in C57BL/6 and BALB/c mouse strains

There were no strain differences in 1- and 3-month-old animals and, as expected, animals of both strains gained weight with age. However, C57BL/6 mice gained weight faster but plateaued earlier than their BALB/c counterparts. As a result, 5 and 10 month old C57BL/6 mice were heavier (P < 0.05) than BALB/c mice (30.4 ± 0.95 and 31.1 ± 0.42 g, versus 27.4 ± 0.83 and 30.5 ± 0.20 g, respectively). At the age of 18 months, due to the continued rise of body weight of the BALB/c strain (34.3 ± 0.47 g) and the plateau of the C57BL/6 strain (31.8 ± 0.97 g), the BALB/c mice were heavier (P < 0.05).

Spleens of BALB/c were consistently heavier than the C57BL/6 mice's spleens. For example, 3- and 5-month-old mice's spleens weighted 3.6 + 0.14 vs. 2.2 + 0.09 and 3.0 + 0.17 vs. 1.8 + 0.17 g/kg BW; BALB/c vs. C57BL/6, 3- and 5-month olds, respectively. With age, relative spleen weight decreased in both strains with the most prominent decline occurring in the C57BL/6 mice from 1 to 3 months (27% decrease: from 3.0 + 0.21 to 2.2 + 0.09 g/kg BW).

### Changes on the number of PBMC and splenocytes in C57BL/6 and BALB/c mice at selected ages

In addition to all phenotypic differences due to strain and age (described below), we also evaluated the effect of strain on the number of PBMC and splenocytes in 3- and 5-month old mice. In accord with the spleen weight data, BALB/c mice had more splenocytes than C57BL/6 mice. In both strains, a moderate decrease in the number of splenocytes was observed as the animals aged from 3 to 5 months (1.8 × 10^7 ^vs. 0.9 × 10^7 ^and 1.6 × 10^7 ^vs. 0.8 × 10^7^; cells/spleen, BALB/c vs. C57BL/6, 3- and 5-month old mice, respectively). In circulation, the difference between the two strains was present in both 3- and 5-month old mice. In addition, a moderate increase in the number of circulating PBMC was observed in both strains from 3 to 5 months of age (5.1 × 10^6 ^vs. 3.5 × 10^6 ^and 6.0 × 10^6 ^vs. 4.2 × 10^6^; cells/ml, BALB/c vs. C57BL/6, 3- and 5-month old mice, respectively).

### Effects of age and strain on the cells of adaptive and innate immunity

#### B cell-specific molecules expression

**Figure 1:**
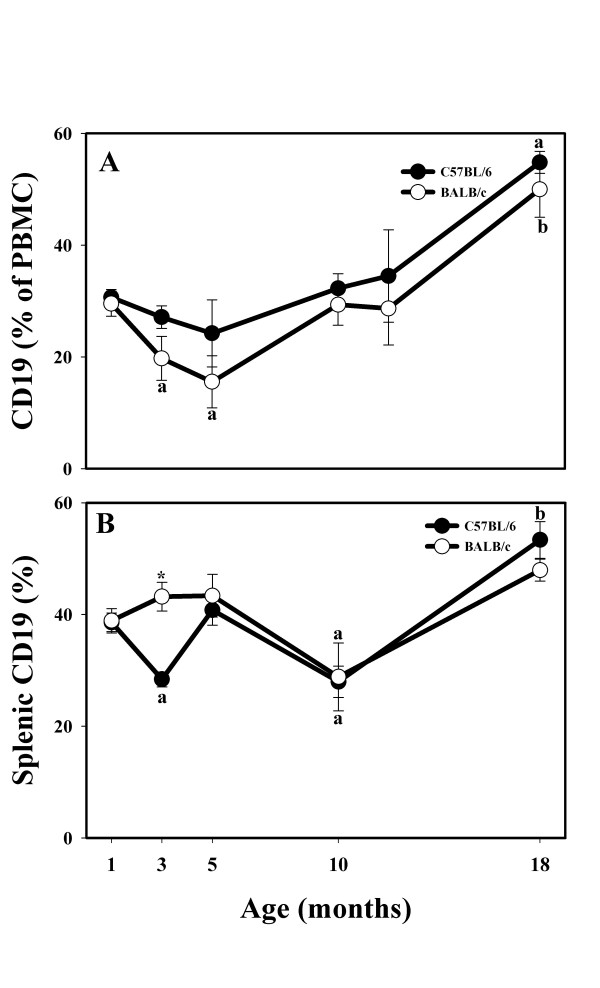
**Effects of age and strain on circulating (**A**) and splenic (**B**) CD19+ lymphocytes collected from male C57BL/6 and BALB/c mice up to 18 months of age.** * Indicates strain differences within a particular age category (P < 0.05). ^a,b ^Indicates age differences within a particular strain (P < 0.05).

Percentage of peripheral blood B cells expressing CD19+ cells increased with age in both strains, peaking in 18-month-old animals (Fig. 1A). There was a decline at 3 and 5 months of age, which was significant only in the BALB/c strain PBMC (Fig. 1A). Overall, the PBMC of C57BL/6 mice had more CD19+ B cells than did BALB/c PBMC (Fig. 1A). However, in the spleens, due to differences in the kinetics of CD19 alterations with age, the % of CD19+ B cells in the BALB/c strain was significantly higher than in the C57BL/6 strain at 3 months of age (Fig. 1B).

#### T cell phenotypes

We did not find any significant age- or strain-related differences in the levels of CD3-bearing T cells in the peripheral blood. Although the percentage of CD3+ cells fluctuated with age in both strains, the fluctuations were non-significant and usually occurred in opposite directions (data not shown). The kinetics of CD3 expression in spleen T cells was different from CD3 expression in peripheral blood T cells in both strains, revealing some strain differences. Thus, the C57BL/6 mice had higher % of circulating CD3+ T cells than the BALB/c mice (data not shown). CD3+ T cells declined only in 5 and 18 months old C57BL/6 mice (data not shown).

**Figure 2:**
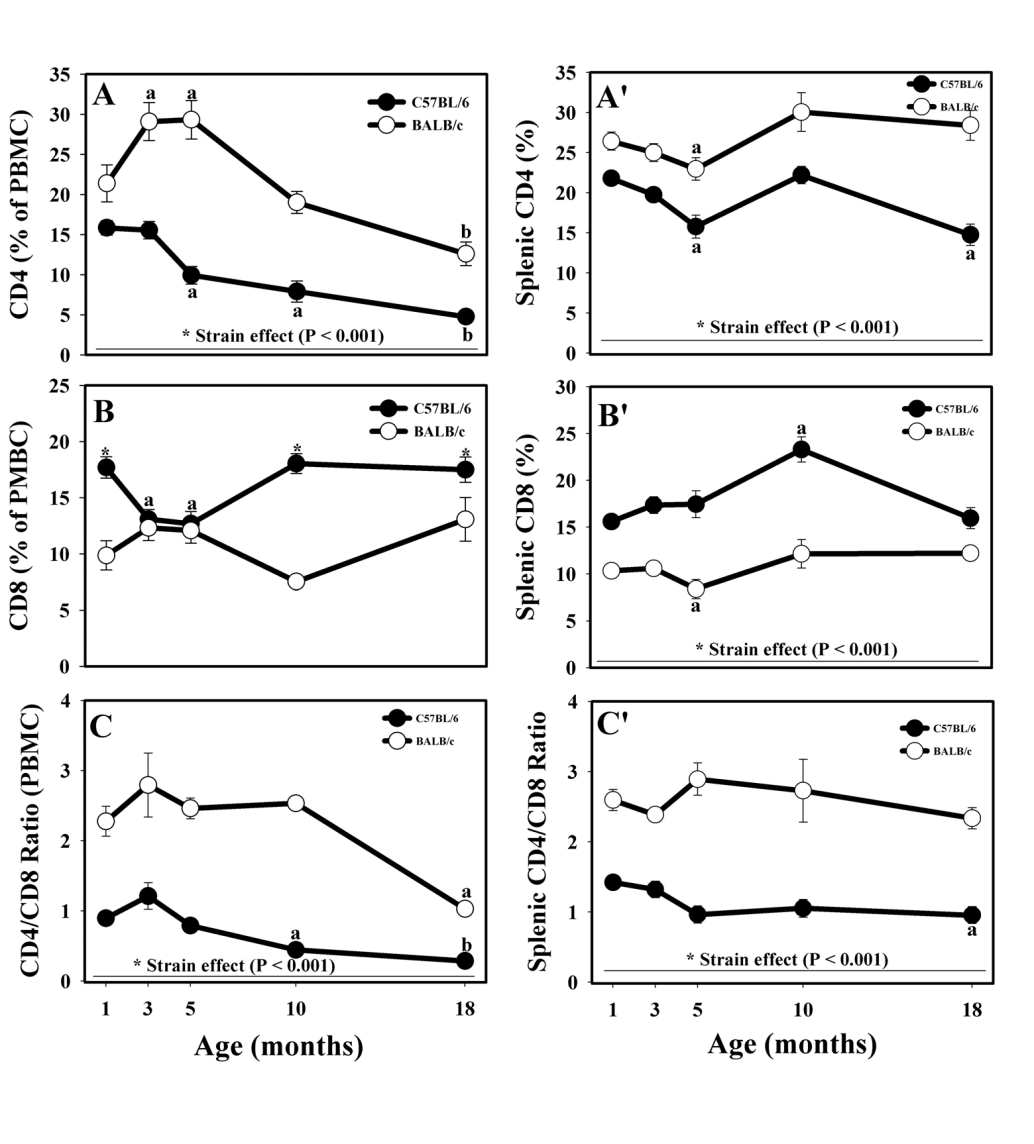
**Effects of age and strain on CD4+ and CD8+ lymphocytes, as well as on the CD4/CD8 ratio in circulation (A, B, and C, respectively) and in the spleen (A', B', and C', respectively) of male C57BL/6 and BALB/c mice up to 18 months of age.** * Indicates strain differences within a particular age category (P < 0.05, or as indicated). ^a,b ^Indicates age differences within a particular strain (P < 0.05).

As expected, BALB/c mice had significantly higher proportions of circulating CD4+ cells than C57BL/6 mice at all ages (Fig. 2A). There were some age-related differences in CD4 expression in BALB/c mice T cells. At the ages of 3 and 5 months, the % of CD4+ cells was significantly higher than the % of CD4+ cells in 1-month old BALB/c mice (Fig. 2A). In both strains, the CD4+ T cells were the lowest in 18-month old mice. However, the decreases in CD4+ T cells occurred earlier and were more pronounced in the C57BL/6 mice (5 months of age) than in the BALB/c strain (10 months of age, Fig. 2A). Similar to PBMC, the % of CD4+ splenocytes was significantly higher in the BALB/c strain than in C57BL/6 mice at all ages (Fig. 2A'). Both strains followed a similar pattern, different from the one in PBMC: moderate decrease up to 5 months of age (significant differences in 5-month-old animals), followed by increase at the age of 10 months and, finally, decline in CD4+ cell numbers in 18-month-old animals that was more pronounced in the C57BL/6 mice (Fig. 2A').

The C57BL/6 mice had higher % of peripheral blood CD8+ T cells than the BALB/c animals at all ages except 3- and 5-month old mice (Fig. 2B). At these ages, CD8+ T cells decreased in the C57BL/6 strain (Fig. 2B). The expression pattern diverged afterwards such that 10- and 18-month-old C57BL/6 mice had more CD8+ cells than BALB/c mice (Fig. 2B). Again, similar to peripheral blood, the C57BL/6 strain had higher % of CD8+ T cells in the spleen at all age groups (Fig. 2B'). There were no striking age-dependent changes in CD8 expression, but there was a significant increase of CD8+ cells in 10-month-old C57BL/6 mice and a significant decrease in the % of cytotoxic T cells in 5-month-old BALB/c mice (Fig. 2B').

Mainly due to the greater % of CD4+ PBMC, the prototypical Th2 mouse strain, BALB/c, had significantly greater CD4/CD8 ratio at all ages (Fig. 2C). Overall, the kinetics of the CD4/CD8 ratio were similar to the kinetics of CD4+ T cells in both strains. Significant age-related drops in the CD4/CD8 ratio occurred at the age of 10 months in the C57BL/6 mice PBMC and at the age of 18 months in both strains (Fig. 2C). As expected, and similar to PBMC, the CD4/CD8 ratio was significantly greater in the spleen of the BALB/c strain throughout ageing (Fig. 2C). The CD4/CD8 ratio was relatively constant at all ages in BALB/c strain having some insignificant fluctuations, whereas the CD4/CD8 ratio decreased beginning at the age of 5 months in the C57BL/6 animals, with the decrease being significant at 18 months of age (Fig. 2C').

In addition to the CD4+ and CD8+ T cells, in the spleens, we investigated the proportion of naïve (CD44^neg/low^) and activated and/or memory (CD44^med/high^) among helper (CD4+) and cytotoxic (CD8+) T cell populations [42-44].

**Figure 3:**
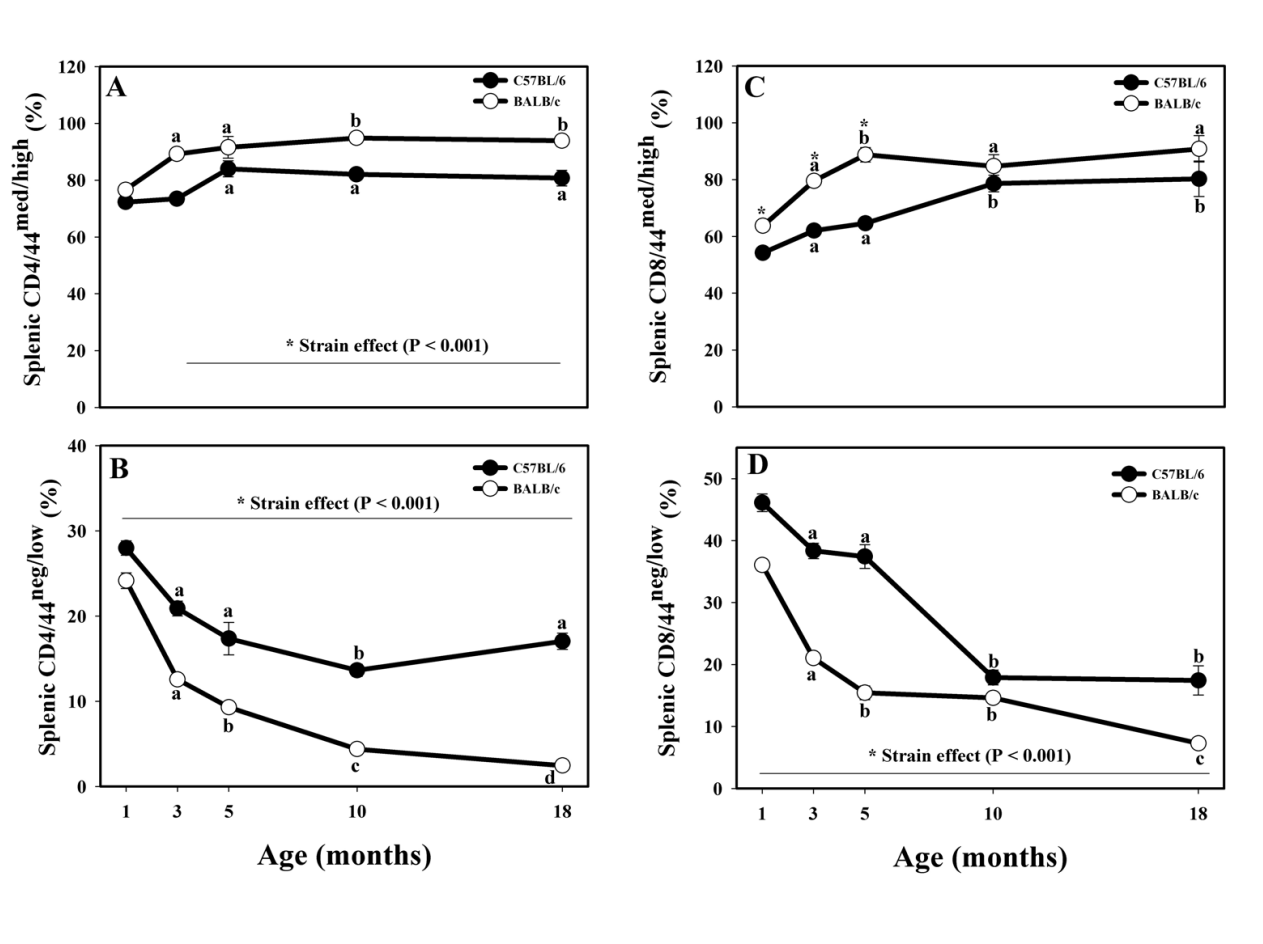
**Effects of age and strain on CD4+/CD44^med/high ^(**A**), CD4+/CD44^neg/low ^(**B**), CD8+/CD44^med/high ^(**C**), and CD8+/CD44^neg/low ^(**D**) splenocytes isolated from the spleens of male C57BL/6 and BALB/c mice up to 18 months of age.** * Indicates strain differences within a particular age category (P < 0.001). ^a,b,c,d ^Indicates age differences within a particular strain (P < 0.05).

The % of CD4+/CD44^med/high ^T cells increased with age in both strains (Fig. 3A), with the increase being more prominent in the BALB/c mice. There were no strain differences in the % of activated/memory CD4+ T cells in one-month-old mice; beginning at three months of age, BALB/c mice had more CD4+/CD44^med/high ^T cells (Fig. 3A).

The percentage of naïve CD4+ T cells (CD44^neg/low^) declined with age in both strains, although the decline in the BALB/c mice was more precipitous (Fig. 3B). C57BL/6 mice had significantly more CD4+/CD44^neg/low ^T cells at all ages (Fig. 3B).

The % of CD8+/CD44^med/high ^cytotoxic T cells was greater in the BALB/c mice up until 5 months of age (Fig. 3C). In both strains, the % of CD8+/CD44^med/high ^cytotoxic T cells increased with age (Fig 3C).

CD8+/CD44^neg/low ^T cells declined with age in both mouse strains (Fig. 3D). The decrease in the BALB/c mice continued at 18 months or age, whereas the decrease in 18-month-old C57BL/6 mice was similar to the one observed in 10-month old C57BL/6 mice (Fig. 3D).

#### Molecules related to professional antigen presentation

**Figure 4:**
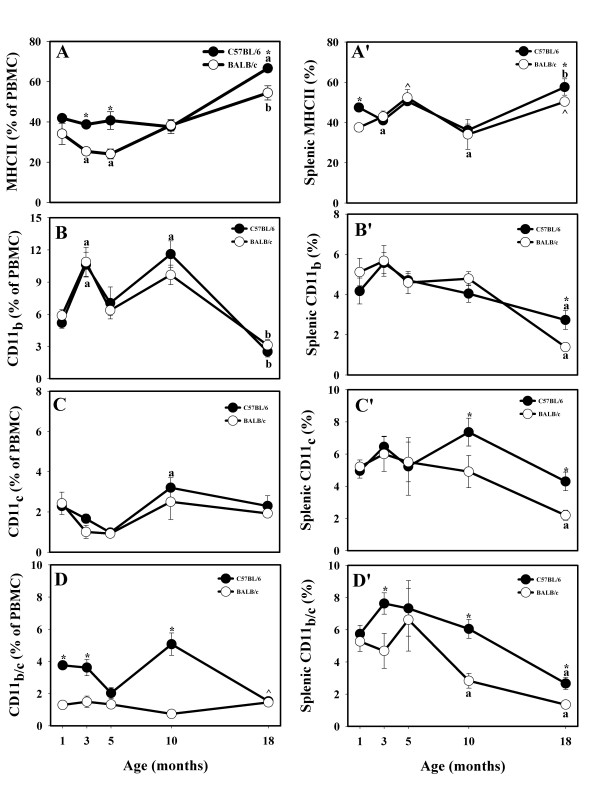
**Effects of age and strain on MHCII+, CD11b+, CD11c+, and CD11b+/CD11c+ positive lymphocytes in circulation (A, B, C, and D, respectively) and in the spleen (A', B', C' and D', respectively) of male C57BL/6 and BALB/c mice up to 18 months of age.** * Indicates strain differences within a particular age category (P < 0.05). ^a,b ^Indicates age differences within a particular strain (P < 0.05). ^^^Indicates P-value of 0.07 (BALB/c).

**Figure 5:**
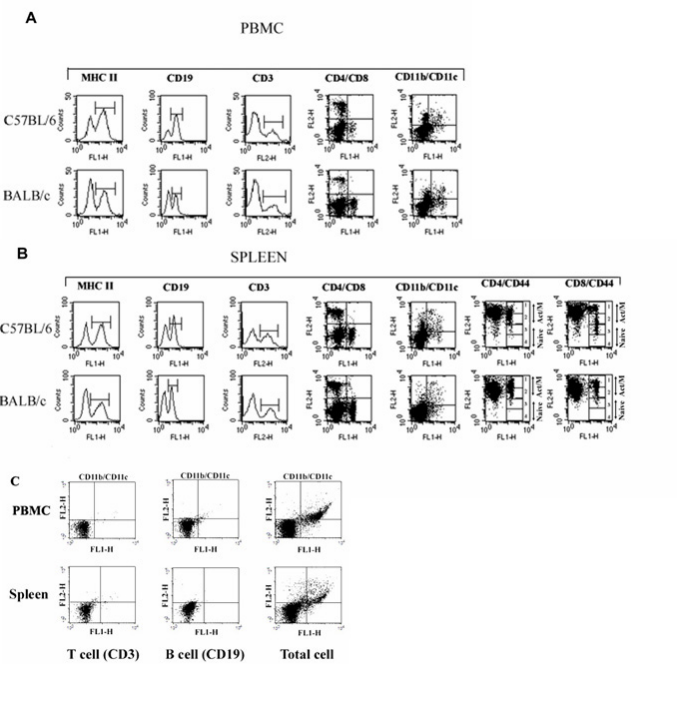
**Flow Cytometry analysis of cell-specific surface molecule expression on peripheral blood and spleen mononuclear cells in C57BL/6 and BALB/c male mice.** Shown on the figure are PBMC (A) and spleen (B) mononuclear cells from randomly chosen mice regardless of age. Cells were separated, stained with directly conjugated mAbs to several cell-specific markers and isotype-matching controls, and gated as low FSC and SSC populations. The MHC class II, CD19 and CD3 staining was analyzed by using single histogram statistics (columns 1, 2, 3, respectively). Two-color analysis for the CD4/CD8, CD11b/CD11c staining was performed by using dot plots with quadrant statistics (columns 4 and 5, respectively). Analysis of the CD4/CD44 and CD8/CD44 staining in the spleen was performed by using dot plots with multiple gate statistics (columns 6 and 7, respectively). The numbers (1, 2, 3, 4 on the dot plots in columns 6 and 7 represent the indicated CD4+ (column 6) and CD8+ (column 7) CD44^high^, CD44^med^, CD44^low^, CD44^neg ^T cell sub-populations, respectively. For statistical analysis, as indicated on (B), the CD44^high ^and CD44^med ^sub-populations were combined into CD44^med/high ^(activated/memory) and, similarly, the CD44^low ^and CD44^neg ^sub-populations were combined into CD44^neg/low ^(naïve) T cell populations. (C) To eliminate the contribution of B and T cells to the % of CD11b/c cells, a three-color analysis (CD19, CD11b and CD11c; CD3, CD11b, CD11c; for B- and T-cell, respectively) was performed and analyzed by dot plots with quadrant statistics.

The % of MHC class II+ cells in peripheral blood increased with age in the animals of both strains, peaking at 18 months (Fig. 4A). There were several age-related differences in the MHC class II+ cells % within each strain. First, in the C57BL/6 strain, the levels of the MHC class II-expressing APC were fairly stable up to 10 months of age (Fig. 4A). Second, there was a significant decrease in MHC class II+ cells up to 5 months of age in the BALB/c strain (Fig. 4A). Due to the decreases of MHC class II molecules at the ages of 3 and 5 months and the more moderate increases at the age of 18 months in the BALB/c strain, the % of MHC II+ cells at these ages was lower in the BALB/c strain than in the C57BL/6 strain (Fig. 5A).

The increases in the % of MHC class II-bearing cells in the spleen were similar to increases in PBMC populations in both strains but were less pronounced (Fig. 4A'). Increases in the level of MHC class II+ cells in 5- and 18-month-old BALB/c mice approached significance (P < 0.07), while 18-month-old C57BL/6 mice had significant increases. The % of splenocytes expressing MHC class II was significantly greater in 1- and 18-month-old C57BL/6 mice than in their BALB/c counterparts, revealing a difference between the two strains (Fig. 4A').

The proportion of CD11b+ PBMC decreased in both strains, with 18-month-old animals having the lowest amounts of CD11b+ lymphocytes (Fig. 4B). Some age-related fluctuations in the % of CD11b+ were apparent in both strains. Thus, the expression of CD11b increased in both strains at the age of 3 months, and only in the C57BL/6 strain at the age of 10 months (Fig. 4B). There were age-related decreases in the % of splenocytes expressing CD11b molecules in both strains, with the lowest % of CD11b+ APC being in 18-month-old mice (Fig. 4B'). There was a strain difference at this age: C57BL/6 mice had significantly more CD11b-bearing APC than BALB/c mice (Fig. 4B').

Variations in the % of APC-expressing CD11c molecules were similar to the variations in CD11b in both circulation and spleen (Figs. 4B, 4C, 4B' and 4C'). The % of double-positive CD11b/c PBMC overall was significantly greater in C57BL/6 mice than in BALB/c mice at all ages except 5 and 18 months (Fig. 4D). Age-related fluctuations in the % of CD11b/c+ cells were more prominent in the C57BL/6 strain than in BALB/c animals (Fig. 4D). In the spleen, the fluctuations in the % of CD11b/c cells followed similar pattern to the fluctuations of CD11b/c cells in the circulation, except that they declined with age in both mouse strains, with the effect being more prominent in the BALB/c mice (Fig. 4D'). To demonstrate that CD11c+ and/or CD11b+/CD11c+ cells in mouse peripheral blood and spleens are primarily DC and their myeloid progenitors, we assessed the adhesion molecule expression in CD3+ T cells and CD19+ B cells in peripheral blood and spleens of 3-month-old mice by using three-color flow cytometry analysis. Both, CD11c and CD11b markers were expressed insignificantly in CD3+ T cells and CD19+ B cells compared to the expression levels in total PBMC or splenic populations (Fig. 5C).

## Discussion

Compared to the T cell compartment, i.e., [41,45], the effects of ageing on professional APC such as B cells, monocytes/macrophages, and dendritic cells (DC) have received much less attention. There is evidence that monocytes and macrophages from aged mice have a reduced functional potential [46,47]. Very little is known about DC changes with age, although their number in the epidermis decreases with age [48,49]. Studies of age-related effects on B cell-mediated immunity are also not as advanced as those of the T cell immune response [2].

Similar to [50], we observed that the expression of MHC antigens on splenic lymphocytes in C57BL/6 mice increases with age. Our data indicated that C57BL/6 animals have higher proportion of B cells at all ages than BALB/c mice. Similarly, previous reports suggests that the relative proportion of B220+ cells is high in C57BL/6 and intermediate in BALB/c mouse strains [8,51]. We also found that the % of CD19+ B cells increases with age in peripheral blood of both strains, while the increases in splenic B cells are prominent in C57BL/6 strain only. This finding, at least for PBMC, differs from earlier reports suggesting that the number of peripheral blood B lymphocytes (B220+) in C57BL/6 and BALB/c mice does not change with age [8,52]. This difference may be due to the different markers used to identify B cells (CD19 versus B220) or to the different sex of the animals (all males used in our study; females in [52]; males and females in [8]).

Our comparison of the kinetics of MHC class II, CD19, and the adhesion molecules CD11b and CD11c expressed on professional APC indicated that, except at the age of one month, C57BL/6 mice had higher % of CD19+ cells. At the same time, we did not observe any strain-related differences in the proportion of CD11b+ or CD11c+ APC in the same age groups. Our data that peripheral blood T and B cells virtually do not express CD11b or/and CD11c are in agreement with the report that the population of peripheral blood CD11b+/CD11c+ APC are predominantly DC [53]. The proportion of CD11b+/CD11c+ APC was significantly greater in 1, 3, and 10 months old C57BL/6 mice than in the BALB/c mice. As previously reported, in general, the proportion of CD11b+/CD11c+ DC in peripheral blood is relatively small [53]. Therefore, increases in the % of DC could not contribute dramatically to the increases in the % of cells expressing MHC class II molecules. Based on the kinetics of MHC class II, CD19, CD11b and CD11c markers in aged animals, we suggest that strain-related differences in MHC class II+ cells were most likely due to the differences in the number of CD11b+/CD11c+ DC in 1 month old mice, CD19+ B cells and DC at the age of 3 months, and CD19+ B cells in 5- and 18-month-old animals.

Unlike in PBMC populations, the spleen cell expression of MHC class II, but not of CD19, was significantly higher in 1-month old C57BL/6 mice. At the same time we did not find any strain-related differences in the % of CD11b+, CD11c+ or CD11b+/CD11c+ APC at this age. Most likely, the CD11b-/CD11c- DC populations contribute to the strain differences at this age. Our data suggest that 18-month-old mice from both strains did not differ in the % of CD19+ B cells. Interestingly, we found significant strain differences in the % of CD11b+, CD11c+ and CD11b+/CD11c+ APC in 18 months old animals. This suggests that the strain difference in the % of MHC class II + APC at the age of 18 months was due to the differences in the % of monocytes and DC [53,54].

In contrast to previously reported data that T cell number increases dramatically with age in mice bred by a cross between CB6F1 mothers and C3D2F1 fathers [55], we conclude that age does not affect the % of total T cells in mouse PBMC from the strains used here. However, differences were evident in splenic T cell populations. C57BL/6 mice had a greater CD3+ T cell % than the BALB/c strain up to the age of 10 months, followed by a substantial decline up to the age of 18 months, resulting in significantly lower % of T cells than their BALB/c counterparts. These results might be explained by the age-related differential accumulation of T cell clones in the spleens of mice from different strains.

Our data are in accord with earlier reports that the prototypical Th2-type strain BALB/c has a greater % of CD4-bearing cells. We observed that at all age groups BALB/c mice have higher % of CD4+ cells than C57BL/6 strain in both PBMC and spleen [8,51]. Similarly, our finding that the % of CD4+ cells decreases with age in peripheral blood regardless of strain agrees with previous reports [17,52,55].

The decline of CD4+ cells with age that occurs in circulation was not observed in the spleens of BALB/c mice. Similar, other groups have found little no or change with age in CD4+ cell proportions in spleen of several inbred mouse strains, including BALB/c [50,56-58]. Yet, in other mouse strains, including C57BL/6, the splenic CD4+ T cells decreased with age [17,52,59], which is what we observed here, albeit of smaller magnitude than in the blood. Thus, CD4+ T cell clonal expansion, which has been described in previous reports apparently occurs with age only in the spleens of the prototypical Th2-type strain BALB/c [17,18].

Peripheral blood CD8+ T cells fluctuated with age in both strains. Fluctuations were greater overall in the C57BL/6 strain, as has been previously observed [8,51]. Our finding that the % of CD8-bearing lymphocytes in peripheral blood does not decrease at the age of 18 months is in accord with previous reports [17,52,18,55]. However, others have reported significant increases in the number of CD8+ T cells in circulation [2,58] or moderate declines in the number of cytotoxic T cells [8].

In general, our results support a gradual, age-dependent shift from naïve CD44^neg/low ^cells towards an increase in CD44^med/high ^cell populations, representing activated or memory phenotypes in mice and humans [8,28,52,55,60,61]. The data presented here are significant regarding these earlier observations in two major ways. First, we found strain differences in the distribution of naïve and activated and/or memory cells in splenic CD4+ and CD8+ T cell populations; BALB/c mice had more activated/memory T-cells and less naïve T cells than C57BL/6 mice. Second, the overall kinetics of CD4+ naïve and activated/memory phenotypes were similar between the two strains and resembled the kinetics of their CD8+ naïve and activated/memory T cells.

Overall, the strain-related phenotypic changes in the splenic APC and T cell populations did not always correlate with the changes in the APC and T cells residing in peripheral blood. There were prominent strain differences in both PBMC and spleen populations in the % of CD4+ (higher in the BALB/c strain), CD8+ T cells (higher in C57BL/6 mice) and CD11b+/CD11c+ APC (higher in C57BL/6 mice). Other strain differences however, were present only in PBMC. Namely, the differences in the % of MHC class II + and CD19+, which were greater overall in the C57BL/6 strain than in BALB/c mice. Of note, the strain difference in the % of CD3+ T cells was only evident in the spleens but not in the peripheral blood. The C57BL/6 strain had greater % of T cells than BALB/c mice. Because of the differences in the composition of the peripheral blood cells and splenocytes [28] spleen cells could not always be used as surrogates for peripheral blood [52].

Age as a factor, influenced phenotypic changes in both strains. There were populations of cells that increased with age in the PBMC and spleens of both strains (i.e., MHC class II+), decreased in the periphery and spleens of both strains (CD11b+) or did not change in the PBMC and spleens of both strains (CD8+). However, in many cases the age-related differences were genetically determined, strongly supporting the evidence of intrinsic connection between genetic background and ageing.

## Conclusion

Taken together, our data provide important information on the principal differences in T cell and professional APC populations between the prototypical Th1 mouse strain C57BL/6 and the prototypical Th2 strain BALB/c. Although many of the age-related changes that occur may be rather subtle and not of much consequence to animals in normal condition, they may become very relevant in conditions of disease and stress. This information might foster development of new strategies to enhance the ability of the immune system to cope with infection at different ages within the context of a particular genetic background.

## Methods

### Animals

Male BALB/c and C57BL/6 mice, 1, 3, 5, 10 (purchased from Harlan, Indianapolis, IN), and 18 months of age (purchased from the National Institute of Aging, NIH, Bethesda, MD) were used in this study. Animals were housed (up to 3/cage) on a 12 h light/dark cycle, with water and food available *ad libitum*. All animal procedures were in accordance with the Animal Welfare Act and the Guide for the Care and Use of Laboratory Animals (NIH publication No. 86-23) and were approved in advance by the Institutional Animal Care and Use Committee (IACUC) of Mississippi State University. At the designated times, animals were sacrificed via CO_2 _asphyxiation, blood was obtained by a cardiac puncture and then immediately transferred into 2 ml vacutainers containing citric buffer (BD Biosciences Pharmingen, San Diego, CA). The tubes were maintained on a rocker platform until peripheral blood mononuclear cells (PBMC) isolation and subsequent analysis. In addition, body weights were recorded; spleens were collected, weighed, placed in 3 ml saline, and maintained on ice until further processing and analysis.

### Cell preparation

#### PBMC

Blood samples were diluted with PBS (1:15), and plasma was removed by centrifugation. To remove red blood cells, samples were incubated with ACK lysing buffer (BioWittaker, Walkersville, MD) for 7 min on ice. Then PBMC were washed twice in PBS and stained with directly conjugated mAbs to several cell-specific markers. PBMC were gated as low forward scatter (FSC) and low side scatter (SSC) populations using Flow Cytometer FACS Calibur (Becton Dickinson, San Jose, CA).

#### Splenocytes

Cell dissociation sieves (Sigma, St. Louis, MO) were used to isolate spleen mononuclear cells. Following dissociation, splenocytes were incubated with ACK lysing buffer for 7 min on ice, washed twice in PBS, and stained with mAbs to different cell-specific markers. Spleen mononuclear cells were gated as described for PBMC.

#### Cell counting

From subset of the samples, an aliquot of the PBMC and splenocyte cell suspensions prepared for flow cytometry was used to determine the number of circulating PBMC and splenocytes. This was accomplished with an electronic cell counter (Coulter Model Z1, Beckman Coulter, Fullerton, CA) as described [62].

### Antibodies and Flow Cytometry

Fluorescein-conjugated mAbs to CD4 (H129.19), CD19 (ID3), MHC class II (28-16-85), CD11b (M1/70), phycoerythrin-conjugated mAbs to CD3 (17A2), CD8 (53-6.7), CD11c (HL3), CD44 (IM7), CD4 (H129.19), Per-CP-conjugated mAbs to CD3 (145-2C11), CD19 (eBIOID3) and isotype-matched controls were used. All conjugated mAbs were purchased from PharMingen/BD Biosciences (San Diego, CA). Isotype-matched controls were purchased from ID Labs (Ontario, Canada). Immunofluorescent staining was analyzed using Cell Quest Version 3.3 Software (Becton Dickinson). The CD19, MHC class II, and CD3 staining was analyzed by using single histogram statistics (Fig. 5A and 5B). Two-color analysis for the CD4/CD8, CD11b/CD11c, staining was performed by using dot plots with quadrant statistics (Fig. 5A, 5B). In the spleen, analysis of the CD4/CD44 and CD8/CD44 staining was performed by using dot plots with multiple gates statistics (Fig. 5B). To eliminate the contribution of B and T cells to the % of CD11b/c cells, a three-color analysis (CD19, CD11b and CD11c; CD3, CD11b, CD11c; for B- and T-cell, respectively) was performed by gating on CD3+ T cells and CD19+ B cells and analyzed by dot plots with quadrant statistics (Fig 5C).

### Statistical Analysis

All cell marker-specific lymphocyte sub-populations were expressed as a percentage of the total PBMC and splenocytes. Then, data was subjected to a two-way (age, strain) analysis of variance (ANOVA). When ANOVA P-value was < 0.05 for a main effect or an interaction, group means were separated by Student-Newman-Keul's multiple comparison *post hoc *test.

## Competing interests

The author(s) declare that they have no competing interests.

## Authors' contributions

LP was responsible for cell separation, immunophenotyping, flow cytometry analysis, manuscript preparation and review. NF was responsible for animal sacrifice, blood and spleen separation, statistical analysis, manuscript preparation and review. Both LP and NF read and approved the final version of the manuscript.
